# PD-1, PD-L1, NY-ESO-1, and MAGE-A4 expression in cutaneous angiosarcoma: A case report

**DOI:** 10.1097/MD.0000000000029621

**Published:** 2022-07-15

**Authors:** Kazuhiko Hashimoto, Shunji Nishimura, Yu Shinyashiki, Tomohiko Ito, Hiroki Tanaka, Kazuhiro Ohtani, Ryosuke Kakinoki, Masao Akagi

**Affiliations:** a Department of Orthopedic Surgery, Kindai University Hospital, Osaka-Sayama City, Osaka, Japan.

**Keywords:** angiosarcoma, MAGE-A4, NY-ESO, PD-1, PD-L1

## Abstract

**Rationale:**

The genomic alteration of cutaneous angiosarcoma (cAS) is complex. Treatment efficacy of immunotherapy for cAS remains controversial and prognosis remains poor. Herein, we report a case of cAS with programmed cell death 1, programmed cell death ligand-1, New York esophageal squamous cell carcinoma-1, and melanoma-associated antigen 4.

**Patient concerns:**

A 69-year-old man presented with a chief complaint of left thumb pain, with a soft tissue mass in the palmar side of the thumb. He had no past medical history. Three months prior, the man experienced the pain while scuba diving. He visited a nearby clinic, and magnetic resonance imaging revealed a soft tissue tumor on the palmar side of the thumb. He was referred to our hospital and a marginal excisional biopsy was performed.

**Diagnosis:**

Pathological findings revealed an angiosarcoma with high-flow serpentine vessels.

**Interventions:**

An excision was performed from the base of the thumb to achieve a wide margin.

**Outcomes:**

One year after the treatment, the patient has not experienced recurrence, metastasis, or complications.

**Lessons:**

Histopathology of the excised specimen was positive for programmed cell death 1, programmed cell death ligand-1, New York esophageal squamous cell carcinoma-1, and melanoma-associated antigen 4; their expression may be a therapeutic target for cAS. Combining immunotherapy with surgical treatment may be effective for cAS.

## 1. Introduction

Angiosarcoma, a malignant vascular neoplasm that variably recapitulates the morphological and immunohistochemical features of endothelial cells,^[1]^ accounts for approximately 3% of soft tissue sarcomas. Angiosarcomas are predominantly found in men, with peak incidence in the seventh decade of life and a wide age range, although they are very rare in children.^[1]^

More than 50% of angiosarcomas occur in cutaneous sites (cutaneous angiosarcoma [cAS]), with the remaining 50% occurring within deep soft tissues, the breast, bone, or viscera.^[1]^

Although there are various treatments for angiosarcoma, including surgery, chemotherapy, and radiotherapy, the prognosis remains poor owing to the aggressive nature of the tumor; moreover, its pathogenesis remains unclear.^[1–3]^

Recently, it was reported that programmed cell death 1 (PD-1), programmed cell death ligand-1 (PD-L1) immune checkpoint mechanism, and cancer-testis antigens, New York esophageal squamous cell carcinoma-1 (NY-ESO-1) and melanoma-associated antigen 4 (MAGE-A4), are involved in the pathogenesis of soft tissue sarcomas (STS).^[4,5]^

We present a case of cAS, which was positive for PD-1, PD-L1, NY-ESO, and MAGE-A4.

## 2. Case report

**Figure 1.**
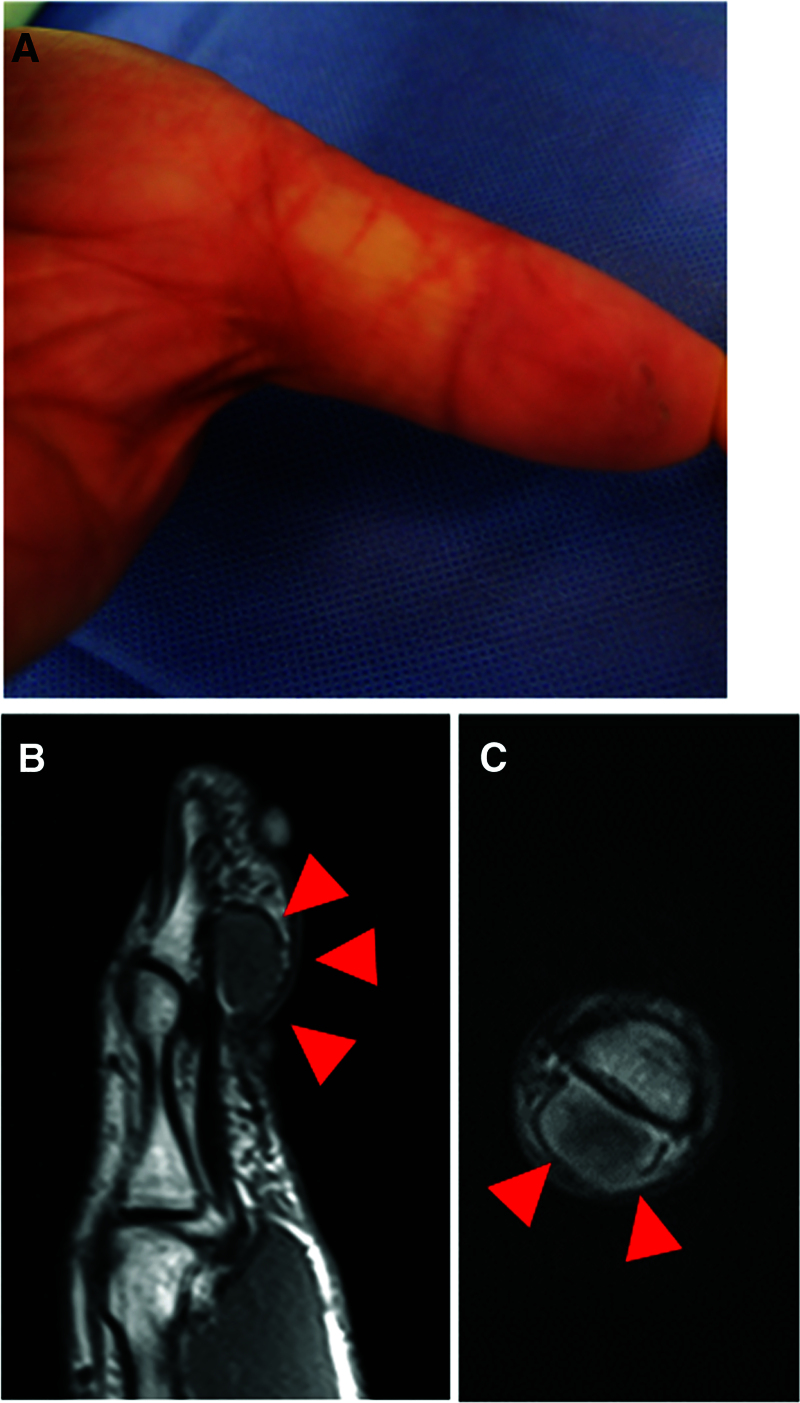
(A) Appearance of the left thumb at the first visit. There is no obvious swelling or abnormal pigmentation. (B) Sagittal T_1_-weighted MRI of the left thumb. A low-intensity mass is observed on the subcutaneous palmar side of the distal thumb (red arrowheads). (C) Coronal T2-weighted MRI of the left thumb. The iso-low-intensity mass is on the subcutaneous palmar side of the distal thumb (red arrowheads). MRI = magnetic resonance imaging.

**Figure 2.**
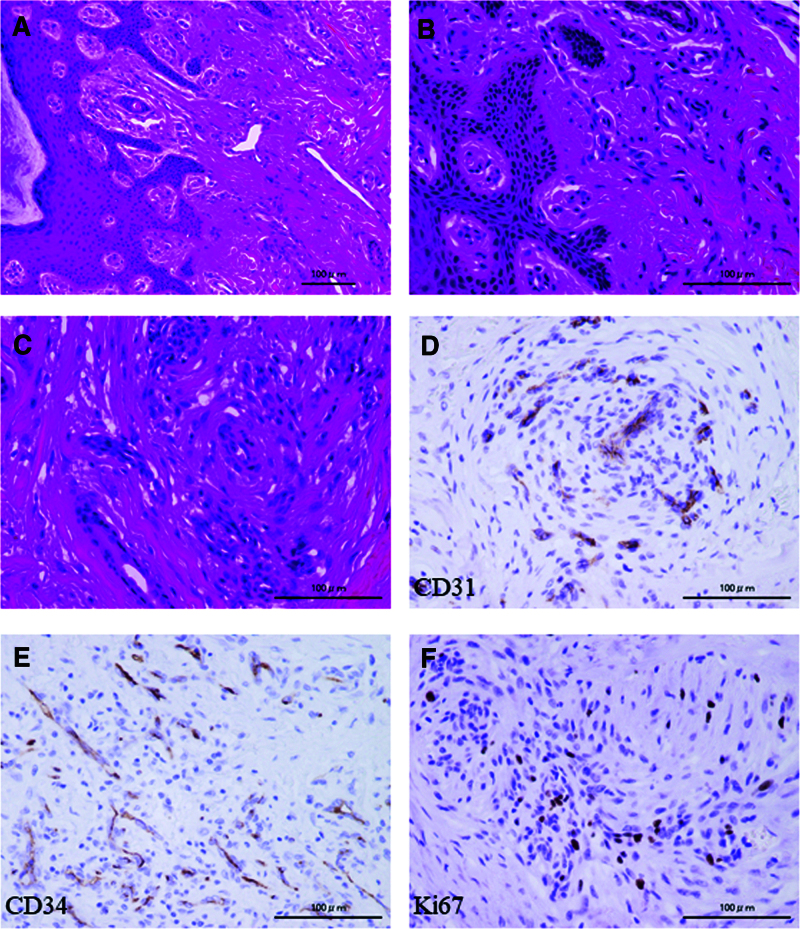
(A, B) Vascular components with atypical endothelial cells are seen in the subcutaneous tissue (A: magnification ×200, B: magnification ×400). (C) There is dense hyperplasia of irregular vascular components, and fibrous tissue is observed in the interstitium (magnification ×400). Scale bars =100 µm. Immunohistochemical findings for CD31 (D), CD34 (E), and Ki-67 (F) (magnification ×400). Positive staining is noted for CD31, CD34, and Ki-67.

**Figure 3.**
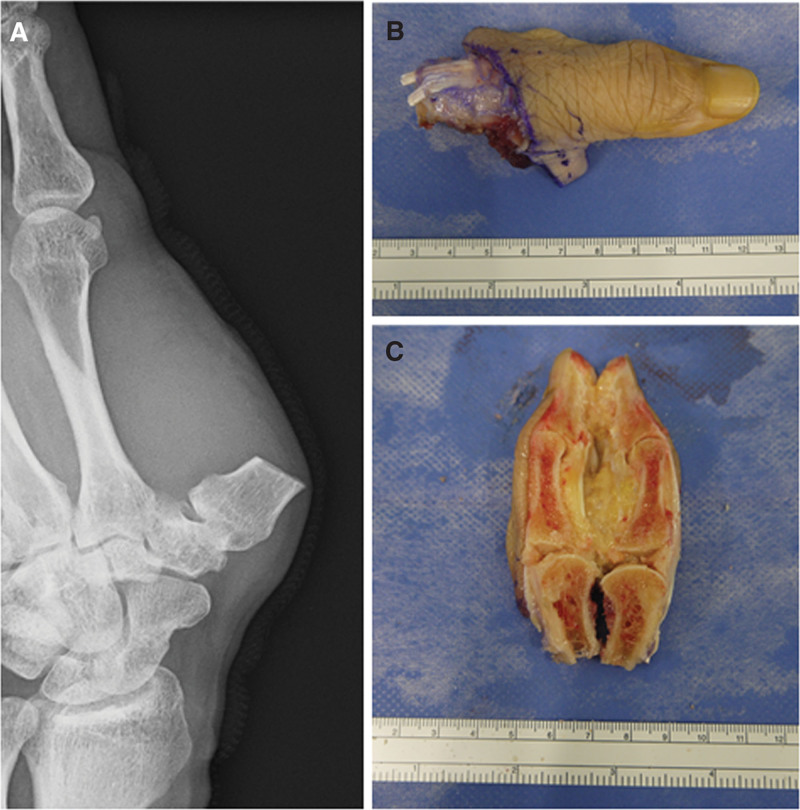
(A) Radiograph of the thumb after surgical treatment. (B) Excised specimen of the thumb. (C) Cut surface of the resected specimen of the thumb.

**Figure 4.**
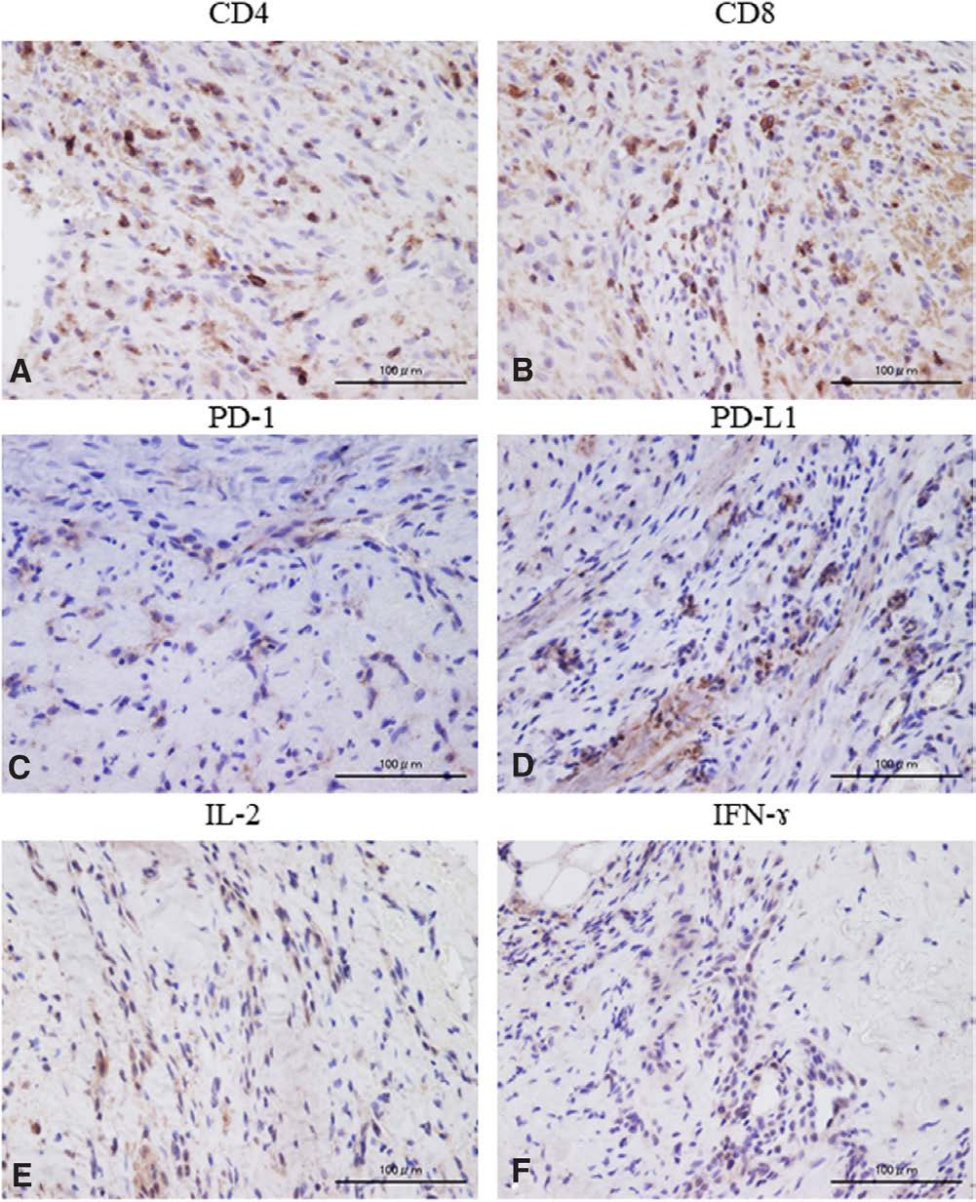
Representative immunohistological positive staining image of CD4 (A), CD8 (B), PD-1 (C), PD-L1 (D), IL-2 (E), and IFN-ɤ (F). CD4 (A) and CD8 (B) positive staining are observed in the tumor-infiltrating lymphocytes. PD-1 (C), PD-L1 (D), IL-2 (E), and IFN-ɤ (F) are positively stained in the membrane and cytoplasm of the tumor cells and lymphocytes. IFN-ɤ = interferon ɤ, IL-2 = interleukin 2, PD-1 = programmed cell death 1, PD-L1 = programmed cell death ligand-1.

**Figure 5.**
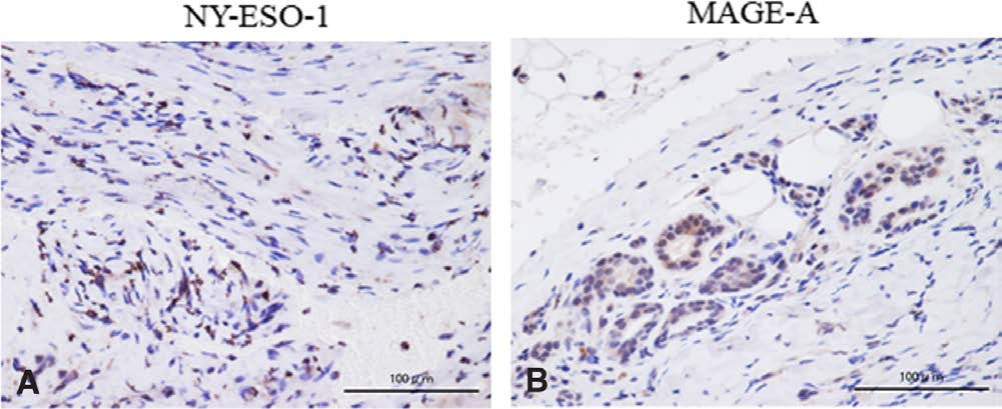
Representative immunohistochemical staining image of NY-ESO-1 (A) and MAGE-A4 (B). NY-ESO-1 (A) and MAGE-A4 (B) are positively stained in the membrane and cytoplasm of the tumor cells and lymphocytes. MAGE-A4 = melanoma-associated antigen 4, NY-ESO-1 = New York esophageal squamous cell carcinoma-1.

A 69-year-old male patient, who had hobby of scuba diving, and dove once a month, presented with a 1 × 1 cm cutaneous mass on the palmar side of the interphalangeal joint of his left thumb, which he had noticed 3 months prior. Owing to the mass growth and pain in that region, he visited a nearby clinic where magnetic resonance imaging (MRI) was performed. He was referred to our hospital because of the finding of a soft tissue tumor. No redness or swelling was observed on physical examination (Fig. 1A). Blood tests did not reveal abnormal findings (data not shown). MRI showed low-signal intensity on T1-weighted imaging and high-signal intensity on T2-weighted imaging, with an inner low-intensity area within the mass (Fig. 1B, C). Marginal resection was performed as an open biopsy procedure. Histological examination of the resected specimen revealed a vascular component augmented by fibrosis (Fig. 2A, B). Dense capillaries, irregular small blood vessels, and atypical cells were also observed (Fig. 2C). Immunohistochemical staining for CD31, CD34, and Ki-67 was positive (Fig. 2D–F). These histological findings confirmed the diagnosis of angiosarcoma, and a wide-margin resection was performed. We resected the thumb, including the thenar muscles, at the level of the proximal metacarpal bone (Fig. 3A). Macroscopic margins were negative (Fig. 3B, C). No lung metastasis was observed on computed tomography (CT; data not shown). Additionally, immunohistochemical staining using test kits was performed for CD4 (rabbit monoclonal, SP35/Roche Diagnostics, Risch-Rotkreuz, Switzerland), CD8 (rabbit monoclonal, C8/144B; Nichirei Corporation, Tokyo, Japan), PD-1 (mouse monoclonal, ab52587; NAT105/Abcam, Cambridge, United Kingdom), PD-L1 (rabbit monoclonal, ab205921; 28-8/Abcam), interleukin (IL)-2 (rabbit monoclonal, ab92381; Abcam), interferon (IFN)-ɤ (rabbit polyclonal, ab9657; Abcam), NY-ESO (mouse monoclonal, E978; Santa Cruz Biotechnology, Santa Cruz, CA), and MAGE-A4 (rabbit monoclonal, ab229011; Abcam), according to the manufacturer’s instructions. Histopathology of the excised specimen was positive for CD4 (Fig. 4A), CD8 (Fig. 4B), PD-1 (Fig. 4C), PD-L1 (Fig. 4D), IL-2 (Fig. 4E), IFN-ɤ (Fig. 4F), NY-ESO (Fig. 5A), and MAGE-A4 (Fig. 5B). The patient provided written informed consent for the publication of the study.

## 3. Discussion

Approximately 50% of cASs occur in the head and neck, especially on the skull of the elderly, and are aggressive malignant tumors with poor prognosis.^[1,6]^ Recently, several molecules, such as FOXM1, HSP90, KCa3.1, miR-497-5p, miR210, and survivin, were identified as potential markers and therapeutic targets for angiosarcoma.^[7]^ In the current report, we presented a case of cAS of the thumb that expressed PD-1, PD-L1, NY-ESO, and MAGE-A4.

Risk factors for angiosarcoma include radiation, exposure to polyvinyl chloride, use of contrast agent, and chronic lymphedema. Notably, 2 previous reports suggested that angiosarcoma arises from pseudoaneurysms.^[8,9]^ Some studies reported that scuba diving causes vascular abnormalities, such as internal carotid artery dissection, stroke, and vascular dissociation.^[10,11]^ In the current case, the patient’s hobby was scuba diving, and this may have contributed to the development of the angiosarcoma.

Angiosarcoma typically presents as a hemorrhagic, diffuse, or multinodular mass of variable size (mean, 5 cm; range, 1–15 cm).^[1]^ In general, the larger the size of the soft tissue sarcoma, the more likely it is to be malignant.^[12]^ In the current study, although the tumor size was relatively small, the angiosarcoma was malignant. Therefore, oncologists should consider malignancy, even for small tumors.

Early diagnosis of soft tissue sarcoma is extremely important for successful treatment and favorable outcomes;^[13,14]^ however, delayed diagnoses of STS and angiosarcomas are common.^[15,17]^ Although MRI findings are relatively nonspecific, they often indicate malignancy and should prompt biopsy for confirmation of diagnosis.^[1,8,9]^ A recent report showed that PD-1/PD-L1, induced by IL-1 and IFN-ɤ, was involved in the pathogenesis of STSs.^[16,17]^ Further, PD-1, PD-L1, NY-ESO, and MAGE-A4 are reportedly useful as diagnostic markers of STS.^[18,19]^ These findings suggest that PD-1, PD-L1, NY-ESO, and MAGE-A4 expression may be useful diagnostic markers for cAS.

Angiosarcomas should be treated with wide-margin resection and adjuvant radiation.^[20–22]^ In the current case, we had planned to perform marginal resection as an open biopsy because the tumor was too small for needle biopsy. Subsequently, we performed wide-margin resection.

Adjuvant chemotherapy is required for patients with unresectable or metastatic lesions.^[6,7]^ Paclitaxel is the standard first-line treatment for advanced angiosarcoma.^[7]^ Second-line treatment options include pazopanib, eribulin mesylate, and trabectedin.^[7]^

Notably, recent reports have shown that immunotherapy may be a therapeutic target for angiosarcoma.^[7,23,24]^ According to recent reports, 30.2% of patient samples were PD-L1 positive, and 17.9% showed high infiltration of PD-1 positive cells.^[25]^ In the univariate analysis, there was a significant relationship between high infiltration of PD-1 positive cells and PD-L1 expression at the tumor site, and favorable survival in stage 1 patients.^[25]^ We also detected PD-1/PD-L1 immune checkpoint molecules that are induced by IL-2 and IFN-ɤ.^[16]^ In addition, NY-ESO-1 expression was observed in 10% of angiosarcomas (2/20),^[26]^ and MAGEA4 was expressed in 41.4% of angiosarcomas, as previously described.^[27]^ In the present case, CD4, CD8, PD-1, PD-L1, IL-1, IFN-ɤ, NY-ESO-1, and MAGE-A4 expression was confirmed. These findings suggest that immunotherapy using combined anti-PD-1/PD-L1 and NY-ESO-1/MAGE-A4 is a potential therapeutic target for cAS.

Angiosarcoma has a poor prognosis, with a reported 5-year overall survival rate of approximately 30%.^[22,28,29]^ The local recurrence rate is approximately 20%, and distant metastases occur in approximately 50% of recurrent cases.^[1]^ The most frequent site of distant metastasis is the lung, followed by the lymph nodes, soft tissues, bone, liver, and other sites.^[1,22]^ Although no lung metastasis was observed in the current case, careful follow-up is necessary.

The present case study had the following limitations: positron emission tomography–CT imaging was not performed; meanwhile, positron emission tomography–CT is useful for tumor staging and response assessment.^[30]^ In addition, 2 genes, protein tyrosine phosphatase receptor type B gene and phospholipase C gamma 1 gene, which were recently identified, have not been investigated.^[31,32]^ Moreover, we could not determine the presence of fusion genes, such as NUP160-SLC43A3, to confirm the diagnosis.^[33]^ Despite these limitations, we confirmed the diagnosis using histological assessment and successfully treated the patient via wide-margin resection.

In conclusion, we reported a case of a tiny cAS of the thumb. We believe that the combined expression of PD-1/PD-L1 and NY-ESO-1/MAGE-A4 may be a useful diagnostic marker and therapeutic target for cAS.

## Author contributions

Conceptualization: K.H., S.N., Y.S., R.K., and M.A.; methodology: K.H., S.N., T.I., H.T., and M.A.; software: K.H. Y.S., H.T., and S.N.; validation: S.N. R.K., K.O., and M.A.; formal analysis: K.H., S.N., T.I., and M.A.; investigation: K.H. Y.S., T.I., H.T., and S.N.; data curation: K.H., Y.S., S.N., T. I., K.O., and M.A.; writing—original draft preparation: K.H., S.N., Y.S., T.I., H.T., K.O., R.K., and MA.; writing—review and editing: K.H., S.N., Y.S., T.I., H.T., K.O., R.K., and M.A. All authors have read and agreed to the published version of the manuscript.

Declaration/disclosure statement: None declared.
